# Acute Graft-Versus-Host Disease: A Brief Review

**DOI:** 10.4274/tjh.galenos.2019.2019.0157

**Published:** 2020-02-20

**Authors:** Elifcan Aladağ, Engin Kelkitli, Hakan Göker

**Affiliations:** 1 Hacettepe University Faculty of Medicine, Department of Hematology, Ankara, Turkey; 2 Ondokuz Mayıs University Faculty of Medicine, Department of Hematology, Samsun, Turkey

**Keywords:** Graft-versus-host disease, Acute, Chronic

## Abstract

Graft‐versus‐host disease (GvHD) is an important complication that can be observed after allogeneic hematopoietic stem cell transplantation (allo-HSCT). Acute GvHD (aGvHD) is seen after allo-HSCT and the incidence of aGvHD is around 30%-50%. aGvHD prophylaxis is essential in patients undergoing allo-HSCT. Initial therapy for aGvHD is steroids. Prognosis is poor in aGvHD patients not responding to steroids. In this article, the pathobiology, clinical findings, prophylaxis, and treatment of aGvHD will be summarized.

## Abstract (tr)

Akut graft‐versus‐host hastalığı (GvHD), allojeneik hematopoetik kök hücre nakli (alloHKHN) sonrasında görülebilen önemli bir komplikasyondur. Akut GvHD (aGvHD) insidansı yaklaşık %30-50 oranında görülmektedir. AlloHKHN yapılan hastalarda GvHD proflaksisi önemlidir. aGvHD gelişen hastalarda başlangıç tedavisi steroiddir. Steroide yanıtsız aGvHD’de prognoz kötüdür. Bu yazıda aGvHD patobiyolojisi, klinik bulguları, profilaksisi ve tedavisi özetlenecektir.

## Acute Graft-Versus-Host Disease (GvHD)

Acute graft‐versus‐host disease (aGvHD) is seen after allogeneic hematopoietic stem cell transplantation (allo-HSCT) [1,2,3,4]. The incidence of aGvHD is around 30%-50% in HLA fully matched allo-HSCT [1]. aGvHD is also common in haploidentical and matched unrelated donor transplantation [1,2].

## Pathobiology

In 1966, Billingham detailed the biology of GvHD development as a three-stage process: a) the graft/donor should contain immunologically competent cells, b) the recipient/host must have tissue antigens not expressed in donor cells, and c) the recipient should be unable to mount an immune response to effectively eliminate the donor cells [3,5]. Hence, during allo-HSCT, after conditioning the host, tissue antigens of the recipient are expressed to the donor T-cells, which leads to donor T-cell activation, expression, and enhanced immune response to the host; in other words, aGvHD occurs [1,2,3,4]. The mechanism underlying tissue damage in aGvHD is massive inflammatory cytokine secretion. Proinflammatory cytokines [tumor necrosis factor (TNF)-α, interleukin (IL)-1β, and IL-6] are seen, as well as the increased expression of the receptor repertoire (pattern recognition receptors) on antigen-presenting cells [6].

## Risk Factors

The most important risk factor for aGvHD is HLA mismatch. Other risk factors include sex disparity between donor and recipient, the intensity of the conditioning regimen, increased age, multiparous female donors, ineffective GvHD prophylaxis, and the source of the graft. A study showed that aGvHD was significantly more common with total body irradiation involving a myeloablative regimen and peripheral stem cell transplantation from a fully matched related donor. In that study, the use of tacrolimus and methotrexate for GvHD prophylaxis was associated with a significant increase in GvHD risk compared to a cyclosporine-methotrexate combination [1].

## Clinical Manifestations

GvHD can be acute or chronic based on the clinical presentation and its occurrence after or before 100 days after allo-HSCT. aGvHD may occur beyond this arbitrary cut-off of 100 days. The widely accepted National Institutes of Health consensus criteria have been used to classify GvHD. GvHD is divided into four subclasses: 1) Classic aGvHD: Diagnostic and distinctive features of chronic GvHD (cGvHD) are absent. Clinical features of aGvHD and present within 100 days of allo-HSCT or donor lymphocyte infusion (DLI). 2) Persistent and/or recurrent late-onset aGvHD: Features of classic aGvHD without diagnostic manifestations of cGvHD occurring beyond 100 days after allo-HSCT or DLI. 3) Classic cGvHD: Present at any time after HSCT. Diagnostic and distinctive features of cGvHD are present without aGvHD. 4) Overlap syndrome: Features of both cGvHD and aGvHD can be seen [4,7].

Clinically significant aGvHD may be cumbersome, affecting both morbidity and mortality [1,2,3,4]. The staging and grading of aGvHD can be seen in Table 1 [4]. The timely diagnosis of aGvHD is important. Hence, numerous novel biomarkers have been recently studied for timely diagnosis. These diagnostic and prognostic markers include systemic biomarkers (microRNAs, suppression of tumorigenicity 2), biomarkers of immune activation [TNF receptor 1,  IL-7, B-cell activating factor (sBAFF)], and organ-specific biomarkers [REG3α (regenerating islet-derived 3-α], S100, TIM (T-cell immunoglobulin domain and mucin domain), cytokeratin-18, hepatocyte growth factor, and skin-derived anti-leukoproteinase, otherwise known as elafin). However, there is no specific GvHD biomarker in routine use [8].

## Prevention

The most important step for the prevention of GvHD is minimizing risk factors with donor selection and a preparative regimen [2,3,4]. GvHD prophylaxis is essential for patients undergoing allo-HSCT [4]. Guidelines for GvHD prophylaxis have been proposed by the European Group for Blood and Marrow Transplantation and European LeukemiaNet [9].

The most common form of GvHD prophylaxis has been the combination of cyclosporine and a short course of methotrexate, which demonstrated improved survival compared to either drug alone. Both cyclosporine and tacrolimus decreased the proliferation of T-lymphocytes [4]. Tacrolimus plus methotrexate is better in decreasing the risk for aGvHD than the combination of cyclosporine and methotrexate, particularly in unrelated HSCT [10]. Both regimens are considered as cornerstones for most GvHD prevention strategies for patients receiving allo-HSCT [11,12]. The effects of the addition of corticosteroids to the combination of cyclosporine and a short course of methotrexate have shown conflicting results [13,14,15]. Calcineurin inhibitors and methotrexate form the main backbone of prophylactic treatment.

## Treatment

The choice of initial therapy for aGvHD depends on the organs involved, the severity of symptoms, and the prophylactic regimen used. Topical steroids are the most commonly used skin-directed therapy for grade I aGvHD. Antihistamines may also be used. Bacigalupo et al. showed that steroid treatment of grade I GvHD prevents progression to grade II GvHD, but not to grade III-IV GvHD [16]. Initial therapy for grade II-IV aGvHD consists of high-dose glucocorticoid steroids. Steroid treatment is effective in approximately half of the patients; those with more severe aGvHD are less likely to respond. Treatment is usually started with the equivalent of 1-2 mg/kg/day of prednisone and then tapered after a decrease in GvHD signs or symptoms. The transplantation-related mortality rate is high in non-responders in the first 5 days of steroid use. Several agents have been added to steroids in comparative studies but no evidence supports the use of these in combination for aGvHD therapy. The best complete response rate was obtained with mycophenolate in combination with other agents (etanercept, etc.) with steroids [17]. Recently the US Food and Drug Administration approved ruxolitinib, a JAK 1/2 inhibitor, and it has been used with considerable success in the treatment of steroid-refractory aGvHD [18].

Unfortunately, there is no standard indication or timing for the initiation of second-line therapy for aGvHD. Many agents have been tested alone or in combination with corticosteroids with limited sustained efficacy [4].

There are few guidelines in the literature regarding second-line cGvHD treatment. Extracorporeal photopheresis (ECP), mycophenolate mofetil, sirolimus, everolimus, rituximab, and ibrutinib are available options. ECP is recommended in the treatment of steroid-resistant aGvHD [19] and was found to result in overall response rates of 50% to 65%.

Table 2 provides a brief summary of some of the current novel second-line strategies for steroid-refractory aGvHD.

## Conclusion

aGvHD leads to significant morbidity and mortality. Therefore, it is crucial to prevent its development. New therapy strategies for both prevention and treatment are needed. aGvHD is a leading cause of late morbidity and mortality. The standard treatment is steroid therapy and a calcineurin inhibitor may also be added. Further treatment strategies need to be developed for the treatment of aGvHD.

## Figures and Tables

**Table 1:**
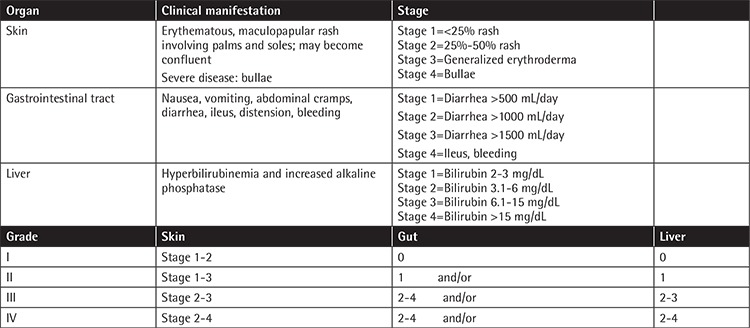
The clinical manifestation, staging, and grading of aGvHD.

**Table 2:**
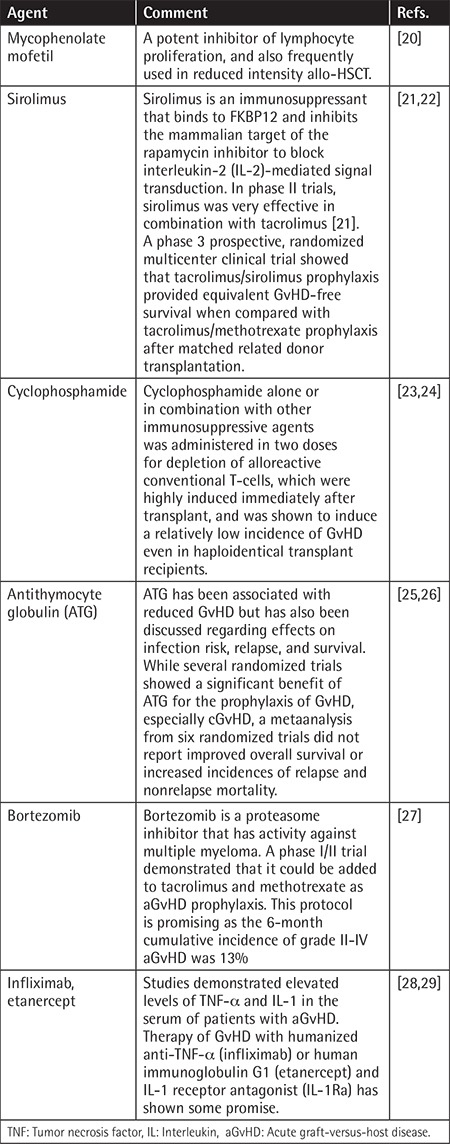
Summary of some of the current novel second-line strategies.
